# Identification and Characterization of an Antifungal Gene *Mt1* from *Bacillus subtilis* by Affecting Amino Acid Metabolism in *Fusarium graminearum*

**DOI:** 10.3390/ijms24108857

**Published:** 2023-05-16

**Authors:** Pei Song, Wubei Dong

**Affiliations:** Key Lab of Crop Disease Monitoring & Safety Control in Hubei Province, Department of Plant Pathology, College of Plant Science and Technology, Huazhong Agricultural University, Wuhan 430070, China; songpei@webmail.hzau.edu.cn

**Keywords:** *Fusarium graminearum*, *Bacillus subtilis*, antifungal gene, transcriptome analysis, amino acid metabolism, branched-chain amino acids (BCAAs)

## Abstract

*Fusarium* head blight is a devastating disease that causes significant economic losses worldwide. *Fusarium graminearum* is a crucial pathogen that requires close attention when controlling wheat diseases. Here, we aimed to identify genes and proteins that could confer resistance to *F. graminearum*. By extensively screening recombinants, we identified an antifungal gene, *Mt*1 (240 bp), from *Bacillus subtilis* 330-2. We recombinantly expressed *Mt*1 in *F. graminearum* and observed a substantial reduction in the production of aerial mycelium, mycelial growth rate, biomass, and pathogenicity. However, recombinant mycelium and spore morphology remained unchanged. Transcriptome analysis of the recombinants revealed significant down-regulation of genes related to amino acid metabolism and degradation pathways. This finding indicated that *Mt*1 inhibited amino acid metabolism, leading to limited mycelial growth and, thus, reduced pathogenicity. Based on the results of recombinant phenotypes and transcriptome analysis, we hypothesize that the effect of *Mt*1 on *F. graminearum* could be related to the metabolism of branched-chain amino acids (BCAAs), the most affected metabolic pathway with significant down-regulation of several genes. Our findings provide new insights into antifungal gene research and offer promising targets for developing novel strategies to control *Fusarium* head blight in wheat.

## 1. Introduction 

*Fusarium graminearum* is a fungal pathogen that infects a variety of cereal crops, including wheat, barley, and maize [1,2]. The disease caused by *Fg* is commonly known as *Fusarium* head blight (FHB) or scab, and it is a significant economic problem for farmers worldwide [3,4]. The FHB reduces crop yield and quality and contaminates grains with mycotoxins including deoxynivalenol (DON) and zearalenone (ZEA), which pose significant health risks to animals and humans [5,6,7]. Antifungal genes are genes that encode proteins that can inhibit the growth of fungi. These genes have been identified in various organisms, including plants, animals, and bacteria. *B. subtilis* is a well-studied model strain known for its antifungal properties. It produces various enzymes, including chitinases, lipases, and proteases, that target fungi. For instance, the SG6 strain of *B. subtilis* secretes lipopeptides that can inhibit the growth of *Fg* mycelium [8], and the ATCC6633 strain produces mycosubtilin, which has been shown to inhibit both the growth and virulence of *Fg* [9]. Additionally, the B3 strain of *B. subtilis* produces iturins and fengycin, both of which are effective inhibitors of *Fg* [10]. In a study by Aktuganov et al., among 70 *Bacillus* spp. strains tested, 19 strains exhibited chitinolytic activity [11]. Until now, research on antifungal genes has been primarily focused on identifying active substances and synthesizing their pathways from isolated antagonistic strains. However, we have employed an alternative approach to obtain antifungal genes or proteins by constructing gene expression libraries. Gene expression library construction and screening techniques were first developed in the early 1980s as fundamental tools for cloning and screening genes [12]. Here, we have utilized gene expression libraries and homologous recombination techniques to screen for antifungal genes in *Fg*.

Ultrasonic cleavage of genomes is a novel and emerging technology that utilizes high-frequency sound waves to disrupt or modify the genetic material of organisms. This technology has shown immense potential in various fields, including medicine, agriculture, and environmental science, as it provides a non-invasive and precise method for manipulating DNA. Ultrasonic cleavage is a new method for obtaining DNA fragments [13,14]. The library preparation for whole-genome sequencing (WGS) using next-generation sequencing (NGS) begins with DNA fragmentation, and sonication is the most commonly used approach due to its ease and reliability [15,16]. In recent years, PEG-mediated protoplast transformation and homologous recombination methods for knocking out and knocking in target genes have become widely popular, and researchers using both can study functional genes, metabolic regulatory networks, and genetic engineering in plants, animals, and microorganisms [17,18,19,20,21]. Gene knock-in allows researchers to study the function of specific genes in fungi as well as engineer fungi for various biotechnological applications [22,23,24]. *Pls1* (FGSG_08695) encodes tetraspanins, which are known to form membrane domains with integrins and signaling proteins. Homologs of *pls1* have demonstrated involvement in the virulence of various fungal species [25,26]. It is noteworthy that the removal of *pls1* does not result in any apparent shortcomings in the growth, development, or virulence of *Fg* [27]. Moreover, regarding overall growth characteristics, tetraspanins do not impact mycelial growth and development or spore morphology and size in the same fungal species [26,28]. Therefore, the *pls1* locus can be used as a target recombination site for studying antifungal genes because its deletion does not affect the normal growth and development of *Fg*.

Amino acids are fundamental constituents of cellular metabolism, serving as precursors for proteins and participating in a variety of metabolic pathways [29]. The metabolic processes governing amino acid biosynthesis in fungi are complex and entail multiple enzymatic reactions and regulatory mechanisms, such as aromatic amino acid biosynthesis and its regulation [30]. Fungal amino acid biosynthesis pathways are similar to those found in plants and bacteria, yet they exhibit a greater degree of complexity due to the existence of multiple pathways for the biosynthesis of the same amino acid and interconnections between different pathways [31]. In *Fg*, amino acid metabolism plays a crucial role in virulence, mycotoxin biosynthesis, and stress responses [32,33,34,35]. Although fungi can obtain amino acids from a range of sources, including exogenous amino acids in their environment, degradation of endogenous proteins, and de novo biosynthesis, the specific mechanisms utilized by fungi to acquire amino acids and the role of amino acid metabolism in nutrient acquisition are intricate and not yet fully understood. In general, amino acid metabolism in fungi is intimately linked to other metabolic pathways, such as nitrogen metabolism and carbon metabolism [36,37]. The mechanisms used by a given fungus to obtain amino acids depend on its ecological niche, nutrient availability, and metabolic capabilities. Under nutrient-poor conditions or when associated with plant hosts with low nitrogen availability, fungi may rely more heavily on de novo amino acid synthesis and recycling of internal amino acid pools to meet their nutritional needs. Conversely, in nutrient-rich environments, fungi may be able to obtain sufficient amino acids through transport from the external environment alone. However, even under these conditions, the metabolic pathways involved in amino acid metabolism are likely to play important roles in regulating growth and metabolism in fungi [38]. 

## 2. Results

### 2.1. Construction of Gene Expression Libraries and Recombinant Screening

**Figure 1:**
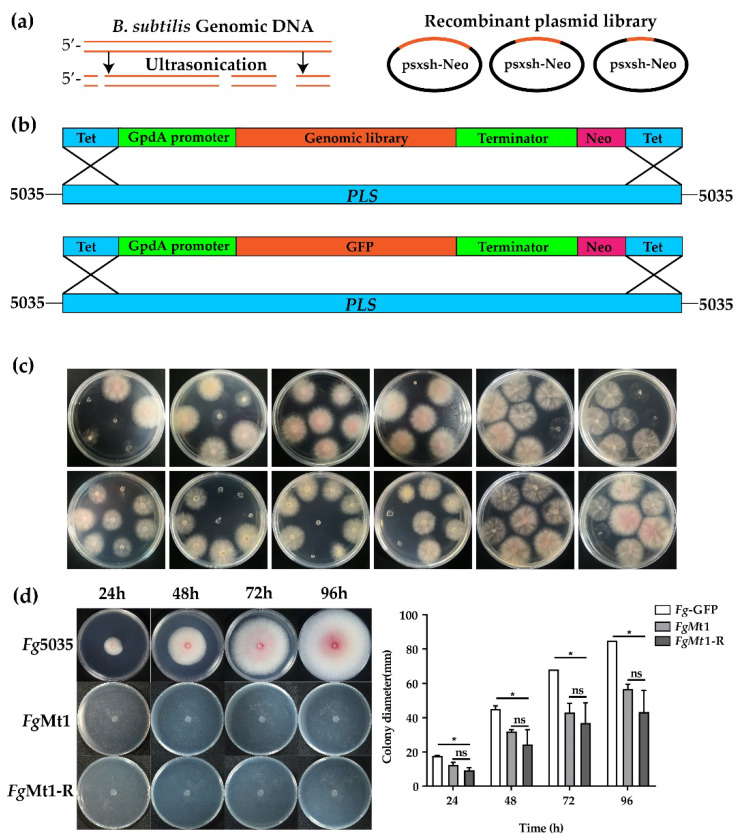
Technical route for the construction of gene expression libraries and recombinant screening. (**a**) Schematic diagram of a randomly interrupted *B. subtilis* genomic library using ultrasonication (left panel), with the interrupted DNA fragments recombined into the psxsh-Neo vector to constitute a recombinant plasmid library (right panel). (**b**) Linearized recombinant plasmid library and GFP gene targeting to the *Fg pls1* locus. (**c**) Examples from the recombinant screening. (**d**) The *Fg*Mt1 strain growth rate was significantly reduced on PDA medium and did not differ significantly from the growth rate of the duplicate recombinants (*Fg*Mt1-R). A *t*-test was used for this experimental analysis, “*” indicates *p* < 0.05, and “ns” indicates no significant difference. The experiment was repeated three times independently, and three single colonies per group were used for the experiment each time.

The endophytic bacterium, *B. subtilis* 330-2, was isolated from rapeseed by our laboratory for the purpose of screening antifungal genes using a gene expression library. To achieve this goal, we first fragmented the genomic DNA of *B. subtilis* (Figure S1a) using ultrasonic cleavage, which ensured random fragmentation of the genomic DNA and allowed for control of fragmentation size and integrity through sonication time, number of sonications, and sonication interval (Figure 1a). After several optimizations, we determined that sonication conditions of 2 s sonication time, 8 s sonication interval, and 30 sonications were optimal for producing DNA fragments ranging in size from 100 to 2000 base pairs, with a concentration between 750 and 1500 base pairs (Figure S1b). We constructed these fragments into a psxsh-Neo expression vector to create a recombinant expression plasmid library. To assess the quality of the library, we randomly selected transformants to verify the recombination rates and fragment size distribution. Our results showed a recombination rate of 89.6% (Figure S1c) and a majority of recombinant fragments ranging from 100 to 1000 base pairs, which is advantageous for screening antifungal genes (Figure S1d). A library of approximately 20,000 recombinant plasmids for subsequent protoplast transformation was obtained.

The gene expression library was introduced into the *pls1* locus of *Fg* wild strain 5035 through protoplast transformation and homologous recombination (Figure 1b). Following screening for phenotypic abnormalities, a strain containing the same GFP gene at the *pls1* locus was generated as a negative control. The observed phenotypic abnormalities, including altered growth rates, abnormal pigment production, and changes in aerial mycelial volume, suggest that the expression of the recombinant genes may impact certain metabolic and regulatory pathways in *Fg* and that the recombinant genes have potential as candidates for antifungal genes. Figure 1c displays some examples screened from the 13,000 recombinants. Notably, a recombinant designated *Fg*Mt1 was obtained during the screening process, which exhibited a significantly reduced growth rate and no aerial mycelium production on PDA medium (Figure 1d). To verify the accuracy and stability of this recombinant, we examined the recombinant site, the insertion of the *pls* site (Figure S2), and the recombinant gene was amplified, reconstructed on the psxsh-Neo plasmid (Figure S3), and retransferred into *Fg-*5035 with the new recombinant named *Fg*Mt1-R, which yielded recombinants with identical phenotypes. The recombinant gene was then utilized to investigate its effect on the transformed strains.

### 2.2. Effect of Recombinant Gene on Recombinant Strain

**Figure 2:**
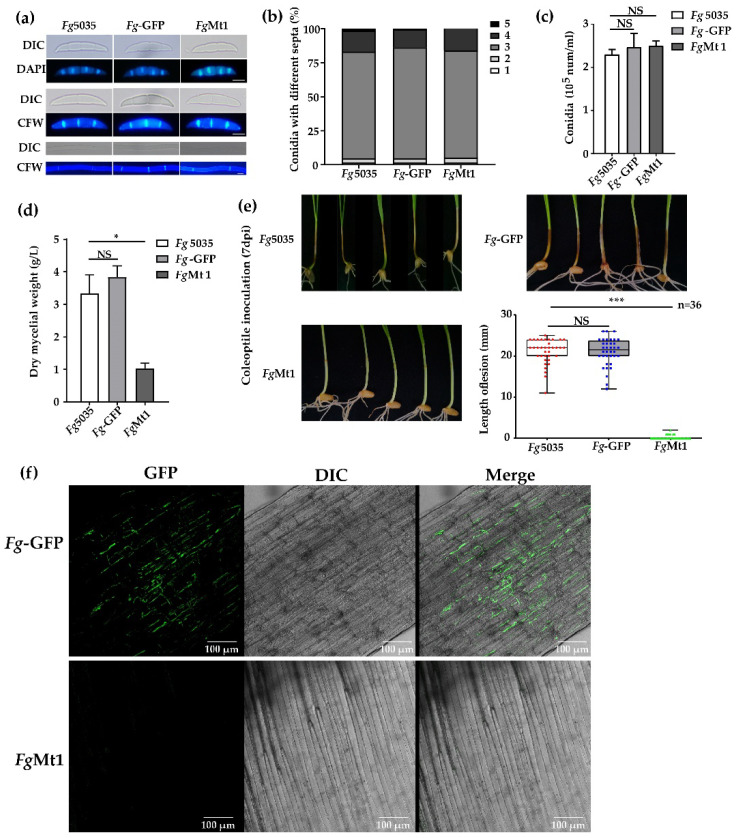
Effects of recombinant genes on recombinants. (**a**) Spore and mycelial morphology of *Fg-*5035, *Fg*-GFP, and *Fg*Mt1; spores stained with DAPI and CFW; mycelium stained with CFW; and bar = 10 μm. There was no significant morphological difference between the above strains. (**b**) There was no significant difference in the spore septum number distribution and corresponding numbers of *Fg-*5035, *Fg*-GFP, and *Fg*Mt1. (**c**) Comparison of the spore production of *Fg-*5035, *Fg*-GFP, and *Fg*Mt1. (**d**) The mycelial production of *Fg*Mt1 was significantly lower than that of *Fg-*5035 and *Fg*-GFP. (**e**) Pathogenicity of *Fg-*5035, *Fg*-GFP, and *Fg*Mt1 spore suspensions (5 × 10^5^ spores/mL) inoculated with wheat coleoptile after 7 days. *Fg*Mt1-inoculated coleoptile was healthy, while wild-type had severe necrosis. (**f**) Fluorescence microscopy of *Fg*-GFP and *Fg*Mt1-inoculated epidermal tissues of the coleoptile, bar = 100 μm. Meanwhile, in the *Fg*Mt1 strain used for (**e**) and (**f**), we transferred a GFP vector used for tracing (Figure S4). A t-test was used for all experimental analyses in this figure, “*” indicates *p* < 0.05, “***” indicates *p* < 0.01, and “NS” indicates no significant difference.

The recombinant *Fg*Mt1 stain obtained by phenotypic screening exhibited abnormal growth on PDA medium with a slow mycelial growth rate and did not produce aerial mycelium. To assess its impact on *Fg*Mt1 hyphae and spore morphology, we stained the septum and nucleus with CFW and DAPI, respectively, and observed them using fluorescence microscopy. Our results showed that the morphology of *Fg*Mt1 mycelium and spores was the same for both the wild-type and negative control *Fg*-GFP (Figure 2a). We further harvested spores from strains *Fg*-5035, *Fg*-GFP, and *Fg*Mt1 after 5 days of growth in CMC spore-producing medium and counted the number of spore septa and spores. No significant difference was observed in the number of spore septa, the number of spores corresponding to the number of septa (Figure 2b), or spore production (Figure 2c), indicating that the recombinant gene did not affect the morphology of mycelium or the morphology and production of spores. However, *Fg*Mt1 exhibited a significant difference in mycelial dry weight compared to the wild type and negative control (Figure 2d). To determine the effect of *Fg*Mt1 on pathogenicity, we used wheat coleoptile inoculation to study its impact. Our results showed that wheat coleoptile treated with *Fg*-5035 and *Fg*-GFP spore suspensions exhibited severe stem disease and browning necrosis, whereas those treated with *Fg*Mt1 spore suspension grew healthy without any disease symptoms (Figure 2e). Statistical analysis revealed that the length of the disease spots in *Fg*-5035 and *Fg*-GFP spore suspension-inoculated wheat coleoptile was significantly higher than that of *Fg*Mt1 spore suspension-treated wheat coleoptile (Figure 2e). Confocal microscopy of wheat stem tissues from the inoculated sites showed that the intercellular spaces of negative control seedling tissues were filled with mycelium, whereas no mycelium was observed in *Fg*Mt1-treated wheat seedling tissues, and the plant tissues remained intact (Figure 2f). Overall, our findings indicate that the recombinant gene had no significant effect on spore and mycelial morphology but had a significant impact on mycelial production and pathogenicity.

### 2.3. Exogenous Additive Partially Restores Recombinant Growth Defects

**Figure 3:**
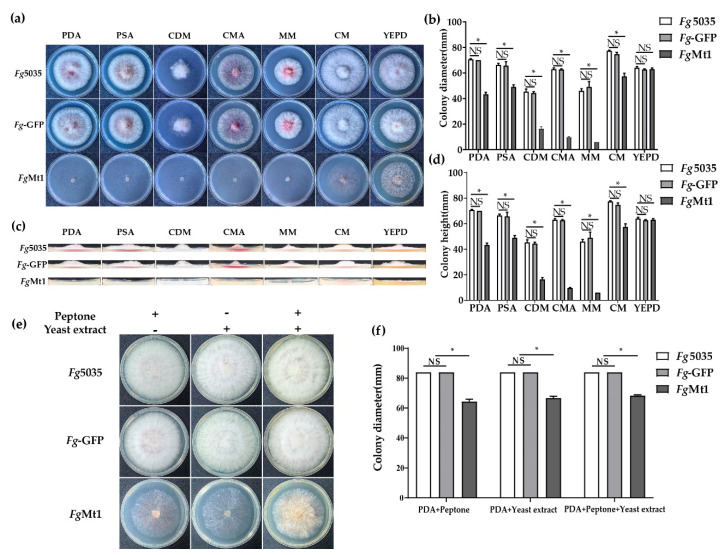
Growth defects and recovery in recombinants. Exogenous additions can restore some of the growth. (**a**) Phenotypes of mycelial morphology of *Fg-*5035, *Fg*-GFP, and *Fg*Mt1 after 72 h incubation on PDA, PSA, CDM, MM, CM, CMA, CAM, and YEPD media. (**b**) Comparison and analysis of mycelial growth rates of *Fg-*5035, *Fg*-GFP, and *Fg*Mt1 after 72 h of incubation on different media. (**c**) Comparative morphology of longitudinal sections of colonies of *Fg-*5035, *Fg*-GFP, and *Fg*Mt1 after 72 h of incubation on different media. (**d**) Comparison and analysis of the colony height of *Fg-*5035, *Fg*-GFP, and *Fg*Mt1 after 72 h incubation in different media. (**e**,**f**) Effects of different nutrient additions (Peptone and Yeast extract) on the growth rates of *Fg-*5035, *Fg*-GFP, and *Fg*Mt1. A *t*-test was used for all experimental analyses, “*” indicates *p* < 0.05, and “NS” indicates no significant difference.

In order to investigate the effect of medium composition on the growth of *Fg*Mt1, we selected seven media with different nutrient compositions (Figure 3a and medium ingredients in Supplementary Materials). We compared the growth rates of *Fg-*5035, *Fg*-GFP, and *Fg*Mt1 on these seven media (Figure 3b). After 72 h of incubation, we found that the growth rate of *Fg*Mt1 on CM medium was significantly higher than that on PDA medium. The growth rate of *Fg*Mt1 on YEPD medium was not significantly different from that of the control, but the aerial mycelium volume was significantly increased. However, *Fg*Mt1 growth remained slow on the other media, and comparison of the growth rates of PDA and PSA showed that sugars were not the main cause of the growth rate difference in *Fg*Mt1. The amount of aerial mycelial biomass was determined by the height of the colony’s longitudinal section (Figure 3c). Only on the YEPD medium did *Fg*Mt1 produce aerial mycelium, which was significantly lower than the control but had a significant increase in mycelial production compared to other media, where no aerial mycelium was produced (Figure 3d). This might suggest that the exogenous addition of certain nutrients plays a role in the phenotypic recovery of the recombinants and might also indicate that these substances have critical roles in the recovery of the recombinant growth defects. To verify that the components in YEPD could restore the slow mycelial growth and the absence of aerial mycelium production of *Fg*Mt1 on PDA medium, although *Fg*Mt1 can produce mycelium, it can’t produce aerial mycelium on the PDA medium compared to the wild type (as shown in Figure 3c), so we added yeast extract and peptone, respectively, to the PDA media (Figure 3e). It can be clearly seen that the growth rate of *Fg*Mt1 mycelium increased in the PDA medium with the addition of both substances (Figure 3f), and although there is still a gap compared to the control, some aerial mycelium could be produced. Such results suggest that exogenous addition of nutrients can restore to some extent the mutant phenotype of *Fg*Mt1 to wild-type, which is most likely due to its own inability to synthesize certain nutrients, and that this phenotype can be compensated for and restored by exogenous addition of some substances.

### 2.4. Transcriptome Sequencing to Analyze the Impact Caused by Recombinant Genes

**Figure 4:**
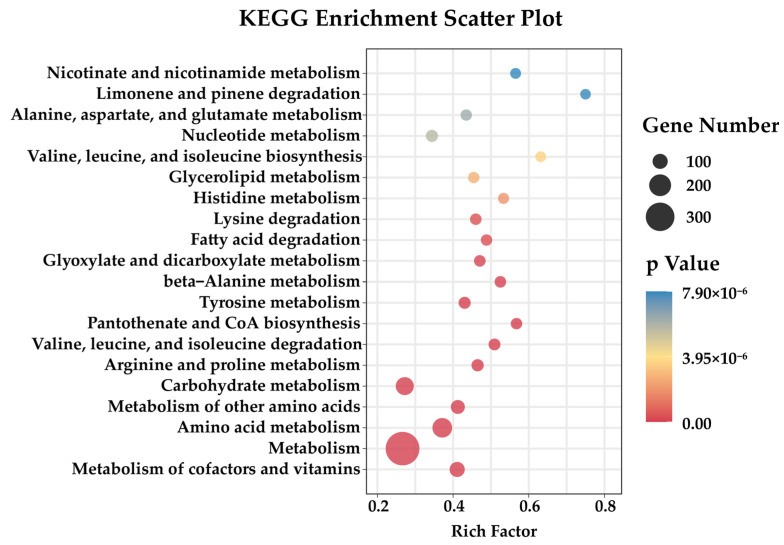
Functional clustering analysis of KEGG-down-regulated genes. The down-regulated genes are mostly enriched in amino acid metabolic pathways, especially branched-chain amino acid metabolic pathways.

To identify how the recombinant gene affects the recombinant at the transcriptional level, we determined the transcriptome of *Fg*Mt1 and analyzed the pathways that the recombinant gene may affect. The wild-type 5035 strain and *Fg*Mt1 were shake-cultured in PDB medium for 3 days, and the mycelia were collected and used to determine the transcriptome. Principal component analysis of the transcriptome data of strains *Fg*Mt1 and *Fg*5035 showed very strong correlation between sample replicates (Figure S6). The correlation heatmap analysis showed high correlation among biological replicates of samples with R-values of 0.995 or greater (Figure S7). Analysis of *Fg*Mt1 recombinant gene expression revealed 1996 genes up-regulated, 1977 genes down-regulated, and no significant difference in the expressions of 5483 genes (Figure S5). The down-regulated genes were found to be enriched in amino acid metabolism pathways, which correlates with the observed phenotypic effects of the recombinant gene on *Fg*Mt1 (Figure 4). The results of transcriptome analysis and phenotypic effects suggest that the recombinant gene may have impacted the amino acid metabolic pathway in *Fg,* leading to down-regulation of related genes, thereby resulting in restricted mycelial growth, limited aerial mycelial production, and reduced pathogenicity. Notably, in the KEGG metabolic pathway analysis, we found that many of the down-regulated genes were enriched in the valine, leucine, and isoleucine degradation and biosynthesis pathways, and they are components of the branched-chain amino acids (BCAAs). The BCAA metabolic pathway is significantly down-regulated in *Fg*Mt1, therefore the growth and development of *Fg* are affected.

### 2.5. Recombinant Gene Sequence Alignment and Bioinformatics Analysis

**Figure 5:**
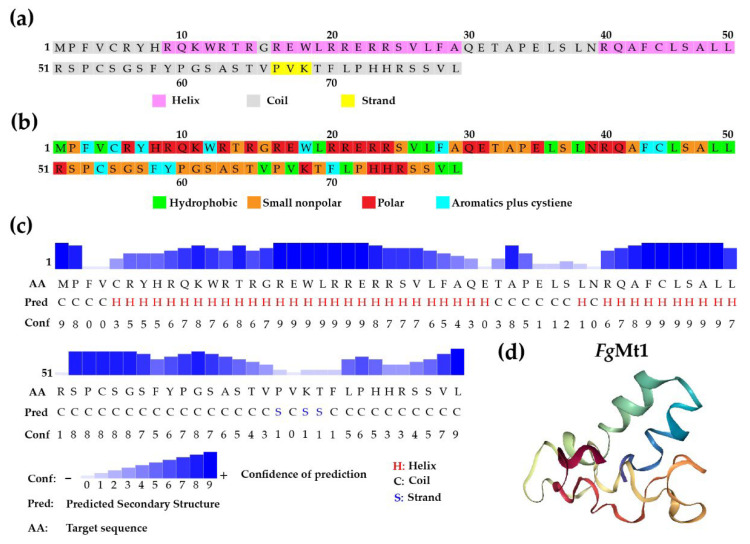
Recombinant gene sequence alignment and bioinformatics analysis. (**a**) Secondary structure prediction of peptide *Mt*1. (**b**) Amino acid hydrophobicity prediction of peptide *Mt*1. (**c**) Confidence analysis of the predicted secondary structure of peptide *Mt*1. (**d**) Visualization of the peptide Mt1 structure. The two helix structures are clearly presented.

The novel antifungal gene sequence *Mt*1 (240 bp) was identified from *B. subtilis* strain 330-2 isolated from rapeseed, which has not yet been fully genomically sequenced. Through comparison with the *Ensembl Bacteria* database, we found that *Mt*1 is homologous to a 234 bp sequence (KS08-04975) in *B. subtilis* str. ATCC 13952 (GCA_000772125) (Figure S11; Table S1). However, the polypeptide encoded by *Mt*1 is distinct from the homologous sequence (AIW33019), making it a novel protein sequence. The 80-amino acid polypeptide *Mt*1 exhibits a multi-segmented helix structure and a segmented strand structure, as predicted using computational methods (Figure 5a,c,d). The structure of the polypeptide is critical for its biological function, as we predicted using Materials Approach 4.6. The secondary structure of *Mt*1 is possibly related to its function and is likely to be the key point of its influence on the metabolic pathway of BCAAs.

## 3. Discussion

The expression of the *Fg*Mt1 gene has a significant impact on the amino acid metabolic pathway in wild-type *Fg-*5035. This impact leads to the down-regulation of many genes related to the pathway. Supplementation of semi-hydrolysis products, such as tryptone or yeast extract, can restore some growth defects, including mycelial growth rate and aerial mycelial production. It appears that the *Fg*Mt1 gene affects the ability of *F. graminearum* to metabolize essential amino acids, which in turn prevents it from synthesizing or utilizing nutrients on its own, forcing the fungus to restore its growth capacity only through exogenous additions. While the mechanism of the effect of the *Fg*Mt1 gene is not yet clear, it appears that *Mt*1 affects the pathway of branched-chain amino acid (BCAA) metabolism in *F. graminearum*. Key genes in this pathway, including FgILv1, FgILv2, FgILv3, FgILv5, and FgILv6, are involved in the synthesis and degradation of BCAAs and significantly affect the mycelial growth and virulence of *Fg* [34,35,39,40]. Knockout mutants of FgILv2 and FgILv6 showed some recovery of the nutritional defects on YEPD medium, consistent with experimental results (Figure 3), and four of five BCAA-related genes were significantly down-regulated in expression in the transcriptome data (Figure S8). These findings suggest that the *Fg*Mt1 gene impacts the BCAA metabolic pathway and affects the growth and virulence of *F. graminearum*.

BCAAs, a group of amino acids comprising leucine, isoleucine, and valine, are essential for protein synthesis and serve as a source of energy and biosynthetic precursors for cellular processes. In fungi, BCAA metabolism plays a critical role in the synthesis of proteins, lipids, and nucleotides, as well as the maintenance of cellular homeostasis [41,42,43]. Fungal growth, development, and stress responses rely heavily on BCAA metabolism, with BCAA catabolism necessary for the utilization of alternative nitrogen sources and for proper mitochondrial function and cellular redox balance. In particular, the leucine biosynthesis pathway (Figure S10) and the *ILV* and *LEU* genes are crucial to BCAA metabolism-related pathways in fungi [44], with FgLEU1 playing a critical role in leucine biosynthesis and full virulence in *Fg* [32]. Rajagopal et al. reported that several key enzymes in the leucine synthesis pathway were encoded by FGSG_12952, FGSG_09589, FGSG_10671, FGSG_06675, and FGSG_09512 in *Fg* [33]. In the *Fg*Mt1 comparison, several genes related to the BCAA pathway were significantly down-regulated in expression (Figure S9), supporting the hypothesis that *Fg*Mt1 has a significant impact on the BCAA pathway in *F. graminearum*.

In *F. graminearum*, the BCAA metabolic pathway is important for the production of virulence factors and secondary metabolites such as mycotoxins, and its breakdown products are substrates for many primary/secondary metabolites [45]. Undoubtedly, the importance of the BCAAs metabolic pathway for the growth and development of *F. graminearum* leads to its use as a target pathway for the study of antifungal proteins and some small proteins that exhibit antifungal activity which were isolated from various organisms, including plants and animals [46,47,48]. Antifungal proteins target various cellular processes in fungi, including cell wall synthesis, protein synthesis, cell membrane integrity, spore production, and production of secondary metabolites [49,50,51,52]. Targeting the metabolic pathway of BCAAs could provide a new approach to the study of antifungal proteins, as it is essential for the growth and pathogenicity of *F. graminearum*. The advantages of *Mt*1 as an antifungal protein for research are: (1) the gene sequence is derived from the *B. subtilis* genome and is extremely easy to obtain; (2) its amino acid sequence is shorter and less difficult to manipulate, whether for secretory expression or optimal modification; (3) it already has candidate target pathways for researches; (4) it is more friendly to the environment as a protein that is easy to degrade. Currently, there are many studies on the active regions of antifungal peptides, including the contribution of core sites to the overall antifungal peptides reported, for example, the β-sheet motif at the C-terminus of defensins, which enhances their antifungal activity [53]; the α-helical structure of Coprisin and Mastoparan B, which enhances their antimicrobial activity [54,55]; and amphiphilic peptides likewise have their unique sites of action to fight against fungi, such as His(2-aryl)-Trp-Arg [56]. It is well known that the structure of a peptide determines its function, and the possible point of action for *Mt*1 to be an antimicrobial peptide is that it possesses two helix structures (as shown in Figure 5a,d), which are predicted with a high degree of confidence (as shown in Figure 5c). Then we have good reasons to believe that the core site of action of *Mt*1 is its helix structure, which determines its function. In subsequent research and drug development, these two structures can also be used as separate objects of study to explore whether they possess the antimicrobial activity and ability of antimicrobial peptides, which will be a very exciting research direction. 

Our results suggest that *Fg*Mt1 may be defective in nutrient acquisition, specifically nitrogen acquisition. Overall, our findings indicate that the recombinant gene inserted in *Fg*Mt1 affects its growth and pathogenicity, likely due to nutrient acquisition defects. These findings contribute to our understanding of the molecular mechanisms involved in the growth and pathogenicity of *Fg* and provide insights into potential targets for future control strategies.

## 4. Materials and Methods

### 4.1. Bacteria, Fungi, and Plant

*B. subtilis* 330-2 was isolated by our laboratory in rapeseed [57], *F. graminearum* 5035 [58] and psxsh-Neo vector [59] were obtained from Prof. Liao (Yucai Liao, from Huazhong Agricultural University), and *Triticum aestivum* L. was obtained from common sources.

### 4.2. Medium and Growth Conditions

All media used in this paper are included here (see Medium ingredients in Supplementary Materials). Lysogeny broth (LB), carboxymethylcellulose sodium medium (CMC), potato dextrose agar medium (PDA), potato dextrose medium (PDB), potato sucrose agar medium (PSA), Czapek-Dox medium (CDM), minimal medium (MM), complete medium (CM), cornmeal agar medium (CMA), carrot dextrose medium (CMA), and yeast peptone medium (YEPD). The *B. subtilis* strain was shaken for 12 h in LB medium, and the *F. graminearum* strains were sporulated by shaking in CMC medium under light at 220 rpm for 5 days and in all the other 7 media for 3 days. The model of shaker used in all the above culture processes is the Ruihua HZ150L constant temperature culture shaker (Wuhan).

### 4.3. Genomic Library Construction

The *B. subtilis* 330-2 strain was grown in LB medium at 37 °C with shaking at 170 rpm/ for 12 h, followed by centrifugation at 9600× *g* for 5 min to collect the cells. Genomic DNA was extracted using the CTAB method after crushing and breaking the mycelium with liquid nitrogen. Ultrasonication was employed to fragment the genomic DNA randomly (2 s ultrasonic treatment and 8 s interval, repeated 30 times) instead of the traditional digestion and ligation reaction method [60]. The resulting DNA fragments were ligated to the psxsh-Neo vector using T4 ligase. The recombinant vectors were transformed into *E. coli* competent cells (TSC 01) and then transferred into *Fg* protoplasts [61,62] to create a mutant library. The *pls1* gene was used as the target site for the integration of the genomic library [59]. To ensure a sufficient number of transformants, the positive ratio and the number of transformants for each *E. coli* transformation were counted, and the number of transformants was confirmed to exceed 20,000.

### 4.4. Coleoptile Inoculation

Wheat seeds were sterilized and prepared for inoculation as follows: Seeds were rinsed with distilled water and then soaked in 75% ethanol for 30 s, followed by three rinses with distilled water. Next, seeds were disinfected with a 2% sodium hypochlorite solution for 10 min and rinsed thoroughly with sterile water. After that, seeds were soaked in sterile water for 2 h at 20 °C. A tray was prepared by placing two layers of wet filter paper on it, and seeds were placed on the filter paper with the embryo facing downward, maintaining a distance between them. The tray was then incubated in a constant-temperature incubator at 20 °C for 3 days until the coleoptiles started to grow. Subsequently, the coleoptiles were inoculated with a spore solution by cutting off the tips of the coleoptiles by 2–3 mm with sterile scissors and then adding 2 µL of the spore solution (5 × 10^5^ spores/mL) to the incision (the control was *Fg-*5035 and the treated group was *Fg*Mt1). After inoculation, the tray was placed in an artificial climate incubator at 25 °C with 12-h light/dark cycles. Symptoms were checked every day, and the tray was moisturized as needed [63].

### 4.5. RNA-Sequencing

*Fg-*5035 and *Fg*Mt1 mycelia were inoculated in PDB medium and incubated for 3 days in the dark at 170 rpm shaking, then the mycelia were filtered, harvested, and frozen in a −80 °C refrigerator for RNA extraction. Total RNA was extracted from *F. graminearum* (wt/treatment) using the TRIzol reagent (Invitrogen, Carlsbad, CA, USA) following the manufacturer’s instructions. PolyA-enriched mRNA was isolated from the total RNA using oligo (dT) magnetic beads, and the RNA was fragmented to an average length of 300 bp by ion interruption. After RNA extraction, purification, and library construction, paired-end (PE) sequencing of the libraries was performed using the Illumina sequencing platform. Sequencing services were provided by Personal Biotechnology Company (Shanghai, China). The resulting data were analyzed using the free online platform Personalbio GenesCloud (https://www.genescloud.cn, accessed on 26 May 2022). Gene Ontology (GO) and Kyoto Encyclopedia of Genes and Genomes (KEGG) enrichment analyses were conducted using the databases established by the Gene Ontology Consortium (http://geneontology.org/, accessed on 14 December 2022) and Kyoto Encyclopedia of Genes and Genomes (http://www.kegg.jp/, accessed on 14 December 2022), respectively.

### 4.6. Bioinformatics Analysis

Recombinant sequences were matched by the *Ensembl Bacteria* database (https://bacteria.ensembl.org/index.html, accessed on 21 February 2023). Protein secondary structure prediction and amino acid affinity analysis website (http://bioinf.cs.ucl.ac.uk/psipred/, accessed on 6 September 2021); protein secondary structure structural confidence analysis; and secondary structure 3D visualization prediction website (https://zhanggroup.org/C-I-TASSER/, accessed on 4 August 2021).

### 4.7. Statistical Analysis

GraphPad Prism version 8.00 (GraphPad Software, San Diego, CA, USA, http://www.graphpad.com/, accessed on 9 March 2019) was used in the statistical analysis. The mean and standard deviation (SD) were used as descriptive statistics. A *t*-test was used for normally distributed variables. All experimental statistics were performed in triplicate, and *p* < 0.05 was considered a statistically significant difference.

### 4.8. *Fg*Mt1 Sequence and Protein Sequence

*Fg*Mt1 gene and protein sequences are available in the Supplementary Material. 

## Data Availability

There is no new data available.

## Supplementary Materials

The supporting information can be downloaded at: https://www.mdpi.com/article/10.3390/ijms24108857/s1.
