# Supplementary material for: Identification and Characterization of an Antifungal Gene Mt1 from Bacillus subtilis by Affecting Amino Acid Metabolism in Fusarium graminearum
Source: Int J Mol Sci. 2023 May 16;24(10):8857. doi: 10.3390/ijms24108857 (PMC10218937; doi:10.3390/ijms24108857)
Supplement: Supplementary file 1 [file ijms-24-08857-s001.zip › ijms-2355636-supplementary.pdf]

Supplementary Materials

Identification and Characterization of an Antifungal Gene *Mt1* from *Bacillus subtilis* by Affecting Amino Acid Metabolism in *Fusarium graminearum*

Pei Song and Wubei Dong

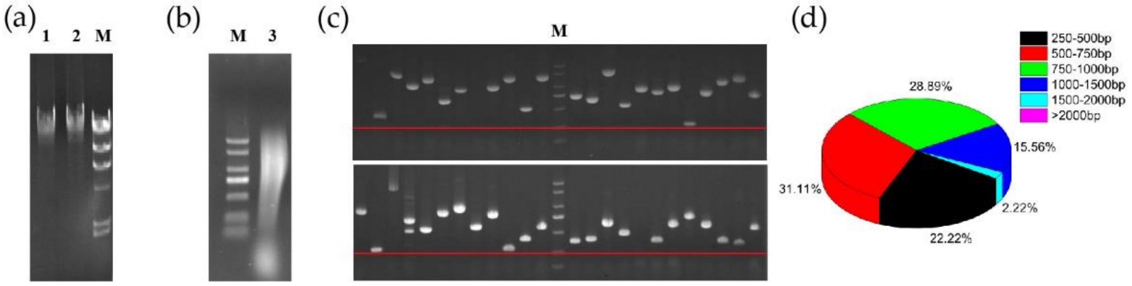

Figure S1. Construction of gene expression libraries. (a) Samples of 1 and 2 are both genomic DNA of *B. subtilis* 330-2, M = λHindIII DNA Ladder Marker. (b) DNA fragment of *B. subtilis* 330-2 genomic DNA by sonication. (c) PCR amplification of recombinant gene, M = DL2000 Marker. (d) Distribution of each group of DNA fragments.

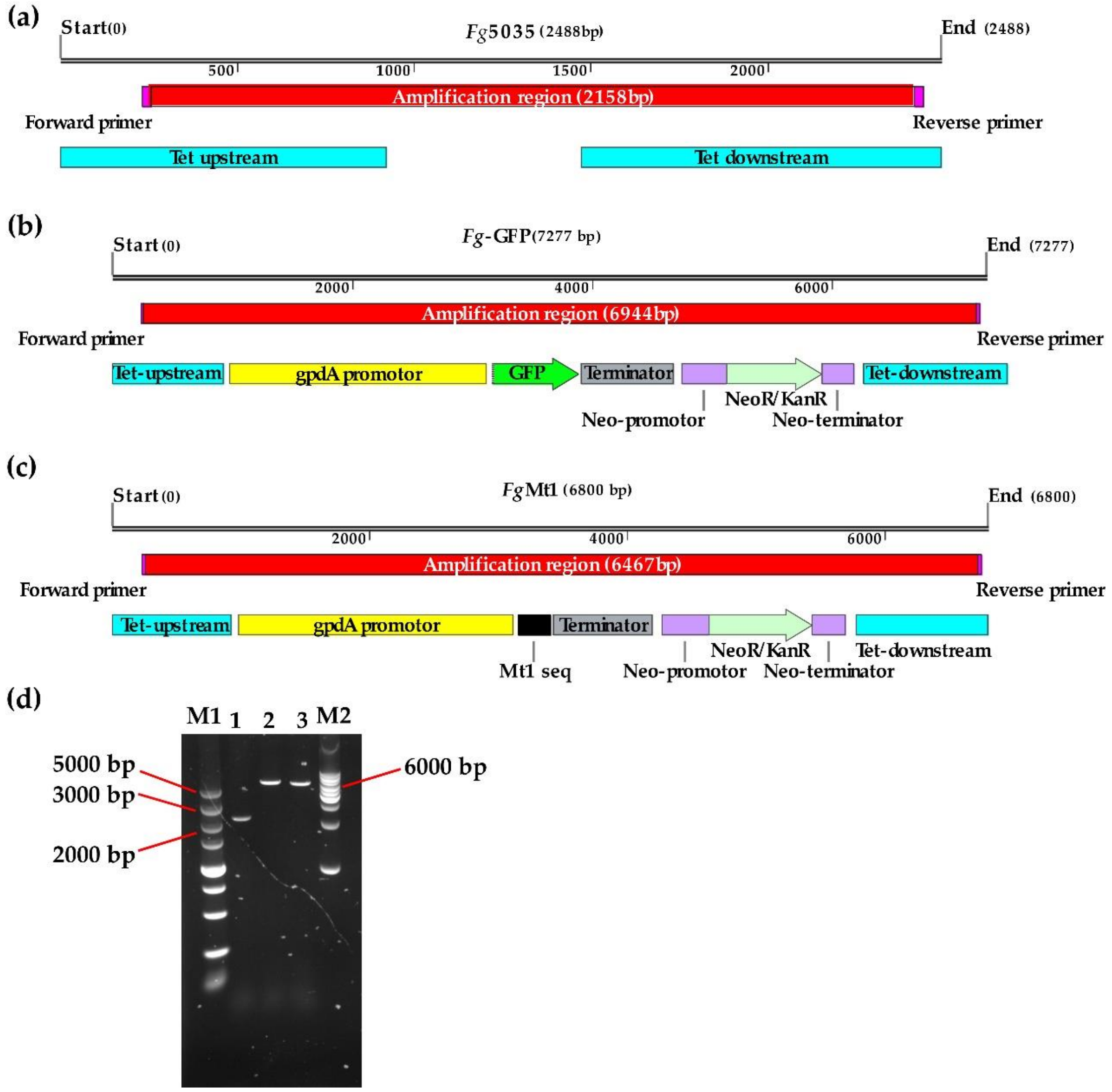

Figure S2. Detection of *pls* locus. (a) Schematic diagram of the genomic *pls* locus of strain *Fg*-5035. (b) Schematic diagram of the genomic *pls* locus of strain *Fg*-GFP. (c) Schematic diagram of the genomic *pls* locus of strain *Fg*Mt1. (d) Detection of *pls* locus insertion by pcr amplification. M1: GL 5000 DNA Ladder; M2: DM 1 kb plus DNA Ladder; 1: Detection of *pls* loci in *Fg*-5035; 2: Detection of *pls* loci in *Fg*-GFP; 3: Detection of *pls* loci in *Fg*Mt1. In addition, the forward primers used for amplification: cctgtaccttaacgcaacactctacc; the reverse primers used for amplification: aagtcacgccataaatcagtcgagaat. Reaction conditions for amplification: (1) 95℃ for 3 min; (2) 95℃ for 15 sec, 74℃ for 10 min, 5 cycles in total. (3) 95℃ for 15 sec, 72℃ for 10 min, 5 cycles in total. (4) 95℃ for 15 sec, 70℃ for 10 min, 5 cycles in total. (5) 95℃ for 15 sec, 68℃ for 10 min, 5 cycles in total. (2) 68℃ for 5 min.

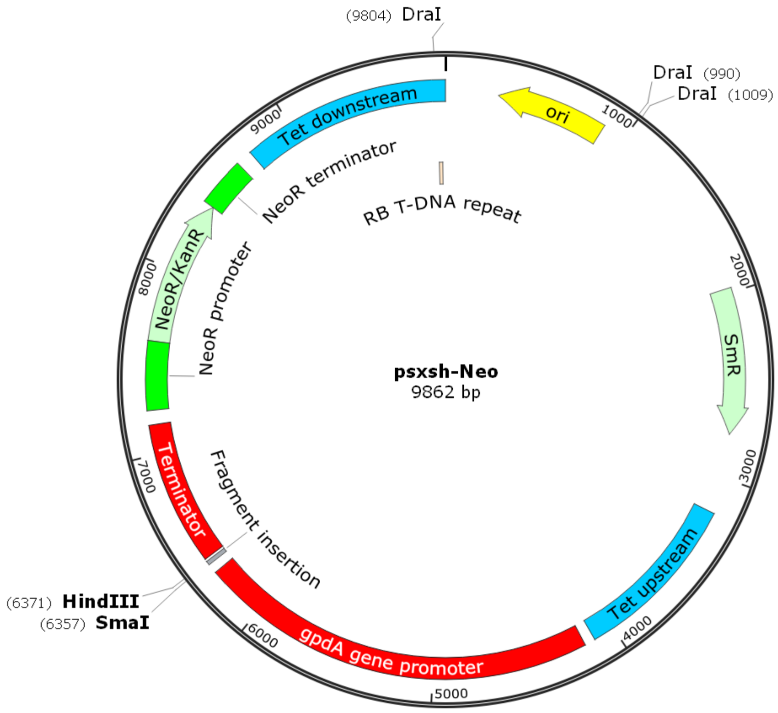

Figure S3. Vector map of psxsh-Neo. The *Dra* Ⅰ restriction enzyme site serves for vector linearization, while the gene fragment insertion site is situated between *Hin*dⅢ and *Sma* Ⅰ. Moreover, the vector includes a gene that confers resistance to neomycin, which is utilized for antibiotic screening.

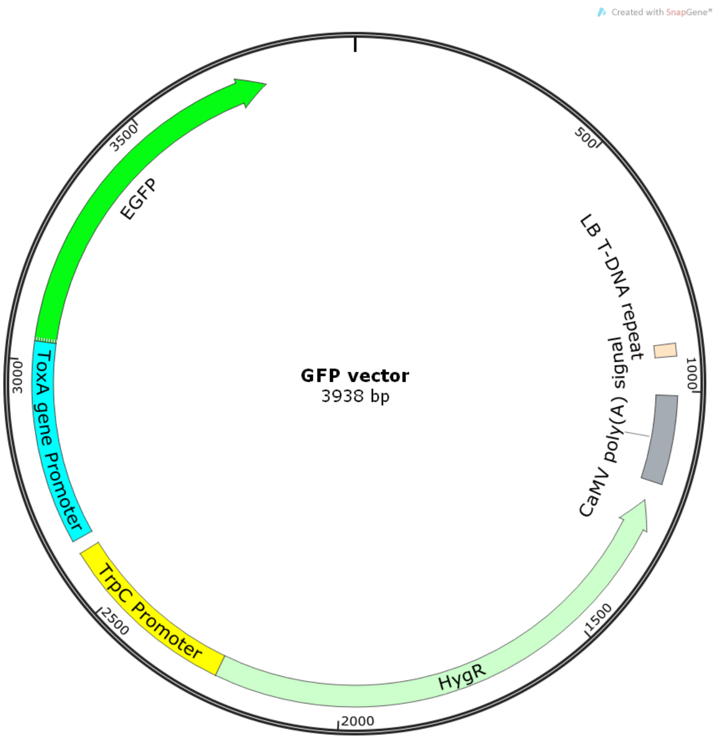

Figure S4. Vector map of psxsh-Neo. The *Dra* Ⅰ restriction enzyme site serves for vector linearization, while the gene fragment insertion site is situated between *Hin*dⅢ and *Sma*

Ⅰ. Moreover, the vector includes a gene that confers resistance to neomycin, which is utilized for antibiotic screening.

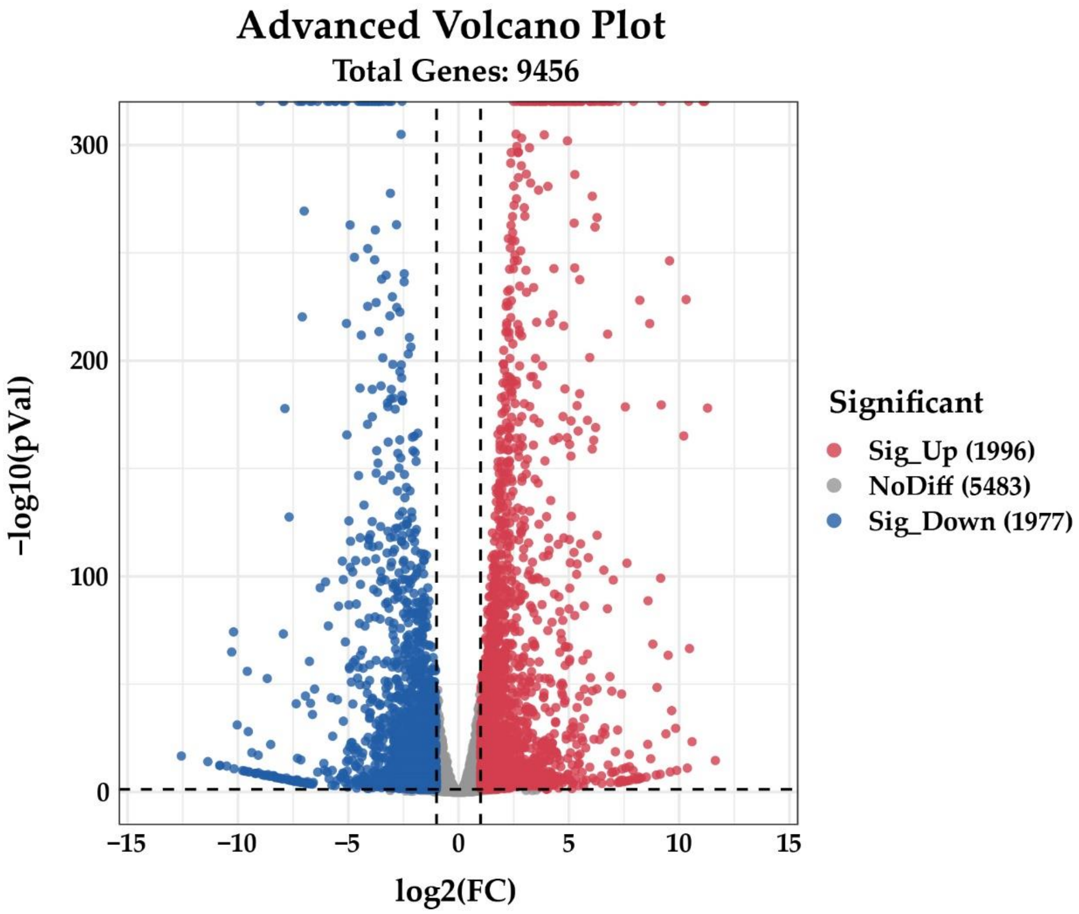

Figure S5. Advanced volcano plot showing gene expression in recombinants, with red dots indicating significantly up-regulated genes and blue dots indicating significantly down-regulated genes.

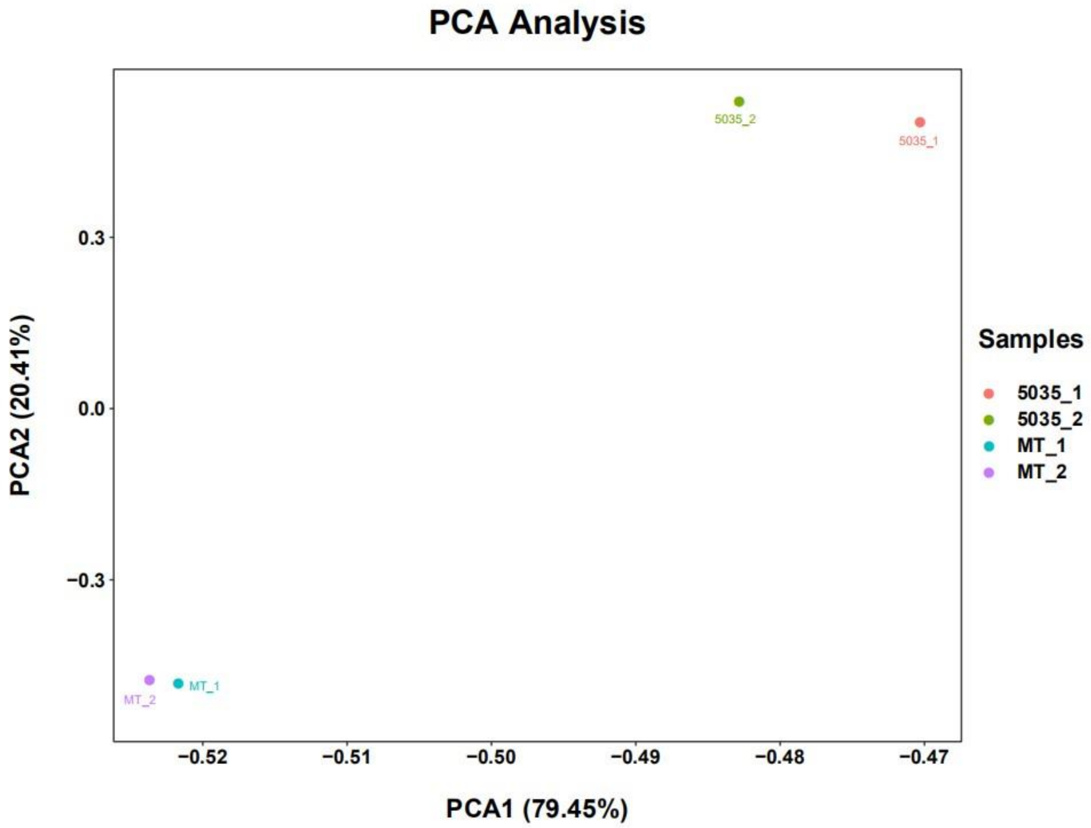

Figure S6. Principal component analysis of transcriptome data of *Fg*Mt1 and *Fg*5035 strains

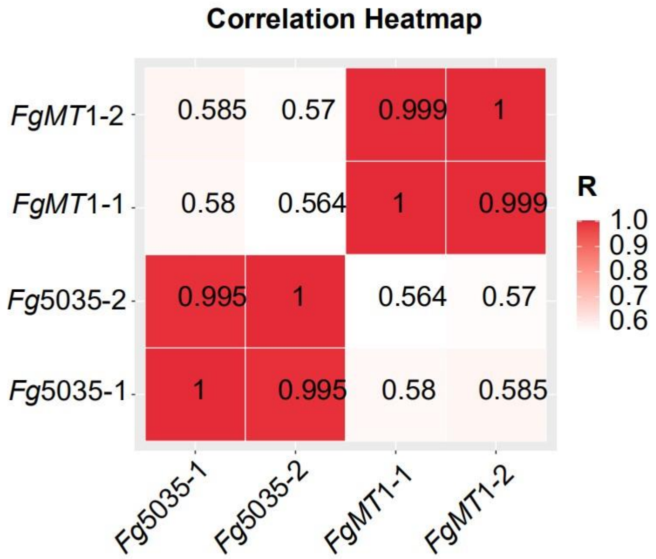

Figure S7. Correlation analysis of transcriptome data between *Fg*Mt1 and *Fg*5035 strain

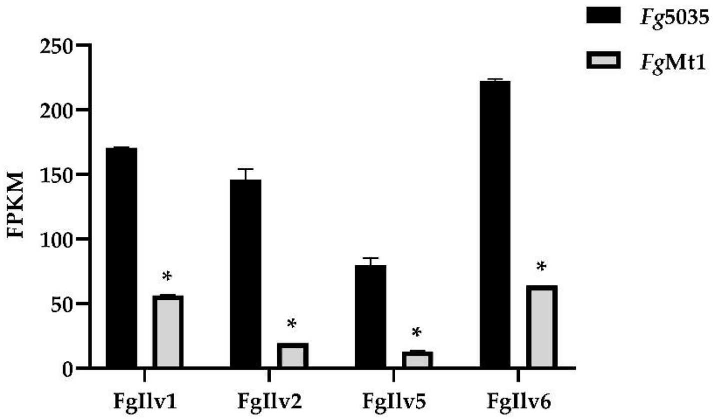

Figure S8. Transcript levels of the FgILV family genes. FgIlv1: FGSG_00296; FgIlv2: FGSG_01086; FgIlv5: FGSG_10118; and FgIlv6: FGSG_06282. T-test was used for the experimental analysis," *" indicates $p < 0.05$ and "ns" indicates no significant difference.

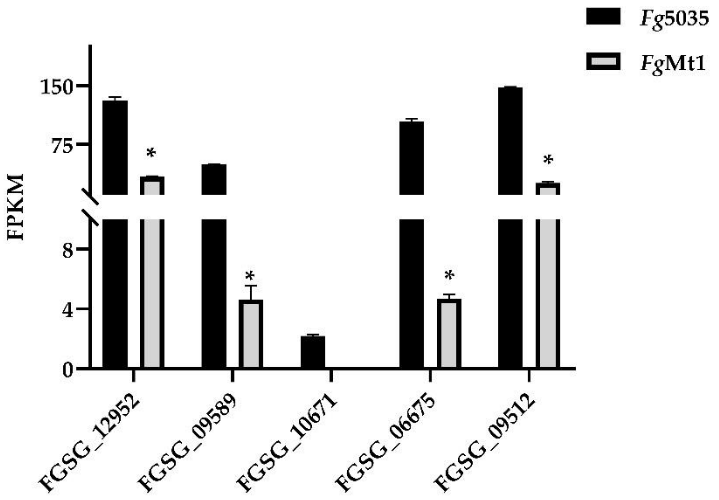

Figure S9. Transcript levels of genes related to the leucine synthesis pathway in *Fg*. T-test was used for thise experimental analysis," *" indicates $p < 0.05$ and "ns" indicates no significant difference.

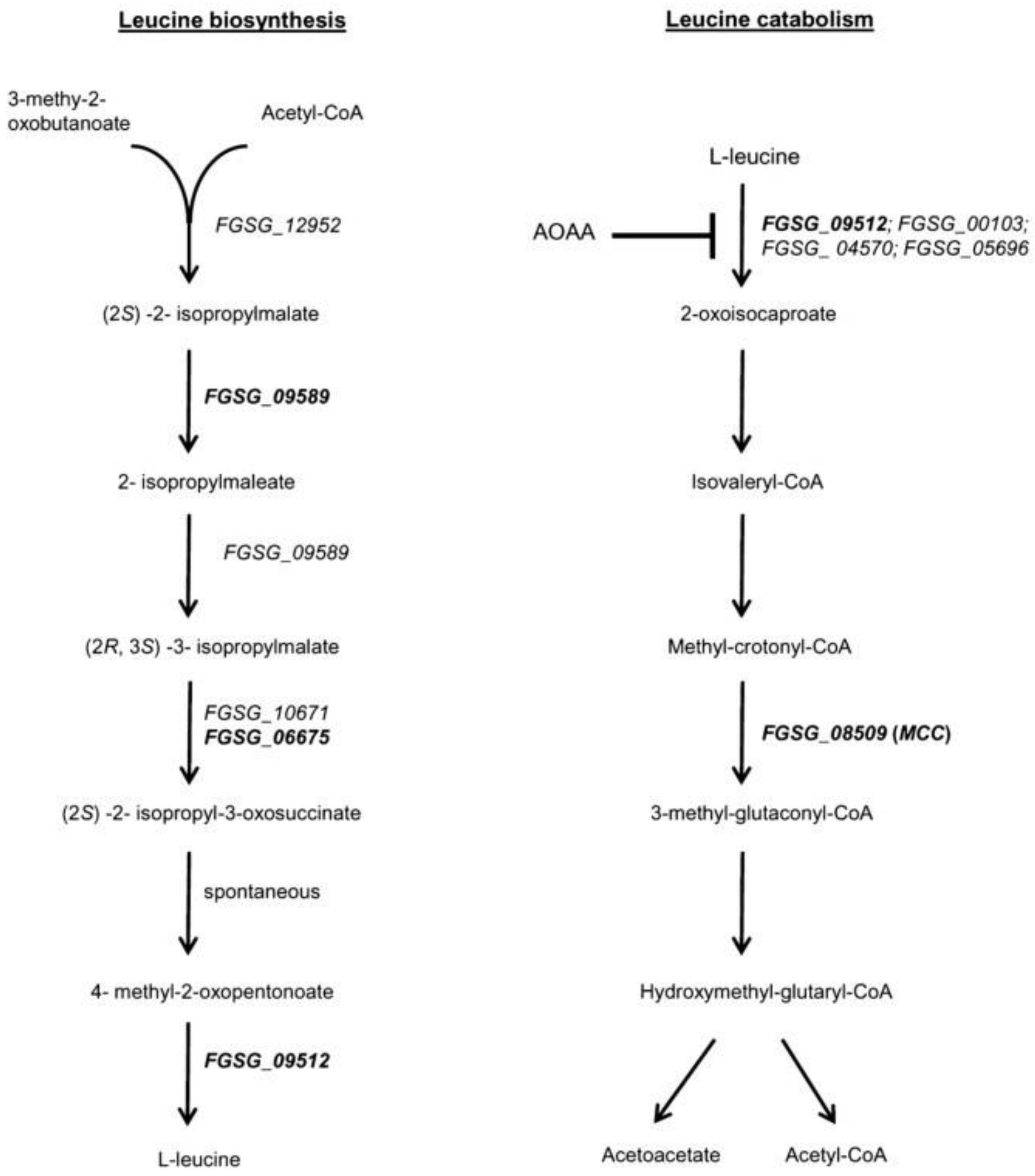

Figure S10. Leucine metabolic pathway in *Fg* [1].

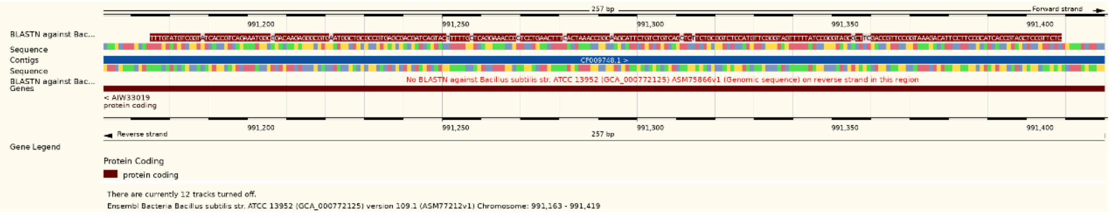

Figure S11. *Mt*1 nucleotide sequence alignment results based on *Ensembl Bacteria* database.

***Fg*Mt1 nucleotide sequence:**

5′-

ATGCCCTTTGTATGTCGGTATCACCGTCAGAAATGGCGGACAAGAGGGCGTGAAT
GGCTGCGCCGTGAGCGACGATCAGTACTTTTTGCTCAGGAAACCGCTCCTGAACT
TTCACTAAACCGGCAAGCATTCTGTCTGTCAGCTCTTCTGCGGTCTCCATGTTCGG
GTAGTTTTTATCCGGGTAGCGCTTCGACCGTTCCGGTAAAGACATTCCTTCCGCAT
CACCGTAGCTCCGTTCTCTGA - 3′

***Fg*Mt1 protein sequence:**

MPFVCRYHRQKWRTRGREWLRRERRSVLFAQETAPELSLNRQAFCLSALLRSPCSGSF
YPGSASTVPVKTFLPHHRSSVL

Table S1. Mt1 nucleotide alignment in NCBI

Genomic Location	Overlapping Gene(s)	Orientation	Length	Score	E-val	%ID
Chromosome:991175-991408	KS08_04975	Forward	234	182	1.50E-100	94.4 [Alignment]
Chromosome:1332247-1332261	KS08_06915	Reverse	15	15	0.68	100.0 [Alignment]
Chromosome:1753259-1753273	KS08_08735	Forward	15	15	0.68	100.0 [Alignment]
Chromosome:2711562-2711576	KS08_13955	Reverse	15	15	0.68	100.0 [Alignment]
Chromosome:241752-241765	KS08_01300	Reverse	14	14	2.7	100.0 [Alignment]
Chromosome:289445-289458	KS08_01540	Forward	14	14	2.7	100.0 [Alignment]
Chromosome:1674652-1674665	KS08_08645	Reverse	14	14	2.7	100.0 [Alignment]
Chromosome:1871441-1871454	KS08_09275	Forward	14	14	2.7	100.0 [Alignment]
Chromosome:2464495-2464508	KS08_12700	Forward	14	14	2.7	100.0 [Alignment]
Chromosome:2893211-2893224	KS08_14950	Reverse	14	14	2.7	100.0 [Alignment]

Medium ingredients

potato dextrose agar medium, PDA (1L)

potato, 200 g

dextrose, 20 g

agar, 13 g

Potatoes peeled and cut into pieces boiled for half an hour, using gauze filter to remove residue, fixed volume to 1L.

potato dextrose medium, PDB (1L)

potato, 200 g

dextrose, 20 g

Potatoes peeled and cut into pieces boiled for half an hour, using gauze filter to remove residue, fixed volume to 1L.

potato sucrose agar medium, PSA (1L)

potato, 200 g

sucrose, 20 g

agar, 13 g

Potatoes peeled and cut into pieces boiled for half an hour, using gauze filter to remove residue, fixed volume to 1L.

Czapek–Dox Medium, CDM (1L)

$NaNO_3$, 3g

KH_2PO_4, 1g

$MgSO_4{\cdot}7H_2O$, 0.5g

KCl, 0.5g

$FeSO_4$, 0.01g

sucrose, 30g

agar, 15~20g

Add ddH2O to 1 L

Minimal Medium, MM (1L)

$NaNO_3$, 2g

KH_2PO_4, 1g

KCl, 0.5g

$MgSO_4{\cdot}7H_2O$, 0.5g

$FeSO_4{\cdot}7H_2O$, 0.5g

sucrose, 30g

Fusarium Trace elements (100mL)
Citric acid, 5g
$ZnSO_4 \cdot 7H_2O$, 5g
$Fe(NH_4)_2SO_4 \cdot 6H_2O$, 1g
$CuSO_4 \cdot 5H_2O$, 0.25g
Add ddH_2O to 1L, adjust pH to 6.9, and add 200μL of Fusarium Trace elements mixture per 1L.

Complete Medium, CM (1L)
20×Nitrate salts, 50mL
Trace elements, 1mL
Vitamin solution, 1mL
yeast extract, 1g
Casamino acid, 1g
peptone, 2g
D-glucose, 10g
After adding ddH_2O to 1 L, adjust the pH to 6.5 with 10 mol/L NaOH.

20×Nitrate salts (50ml)
KH_2PO_4, 1.52g
KCl, 0.52g
$MgSO_4 \cdot 7H_2O$, 0.52g
$NaNO_3$, 6g

Trace elements (1L)
Na_4EDTA, 5g
$ZnSO4 \cdot 7H_2O$, 2.2g
H_3BO_3, 1.1g
$FeSO_4 \cdot 7H_2O$, 0.5g
$MnCl_2 \cdot 4H_2O$, 0.5g
$CoCl_2 \cdot 6H_2O$, 0.17g
$CuSO_4 \cdot 5H_2O$, 0.16g
$Na_2MoO_4 \cdot 5H_2O$, 0.15g

Vitamin solution (1L)
PABA (p-aminobenzonic acid), 0.01g
Nicotinic acid, 0.01g
Thiamine, 0.01g
Biotin, 0.01g
Riboflavin, 0.01g
Pyridoxin, 0.01g

Cornmal agar medium, CMA (1 L)
Cornmal, 40 g
Agar, 10-15 g
Add ddH_2O to 1L.

Yeast extract Peptone Dextrose medium, YEPD (1 L)
yeast extract, 3 g
peptone, 10 g
dextrose, 20 g
agar, 10-15 g
Add ddH_2O to 1L.

Carota agar medium, CAM (1 L)
carota, 200 g
dextrose, 20 g
agar, 10-15 g
Carota peeled and cut into pieces boiled for half an hour, using gauze filter to remove residue, fixed volume to 1L.

1. Subramaniam, R.; Narayanan, S.; Walkowiak, S.; Wang, L.; Joshi, M.; Rocheleau, H.; Ouellet, T.; Harris, L.J. Leucine metabolism regulates TRI6 expression and affects deoxynivalenol production and virulence in Fusarium graminearum. *Mol Microbiol* **2015**, *98*, 760-769, doi:10.1111/mmi.13155.
